# Reviewed article: Cardins KKB, Silva IS, Soares A, Carvalho LMA, Graça JMB, Freitas CHSM. Factors associated with post-COVID-19 health conditions at a rehabilitation center: a cross-sectional study, Campina Grande, Paraíba, Brazil, 2024. Epidemiol Serv Saude. 2026:35;e20250305.

**DOI:** 10.1590/S2237-96222026v35e20250305.a

**Published:** 2026-04-20

**Authors:** Anna Carolina Arena Siqueira

**Affiliations:** 1 Ministério da Saúde, Brasília, DF, Brazil Ministério da Saúde Brasília DF Brazil

## Peer review A

## Questionnaire

Does the manuscript contain new and significant information to justify publication? Yes

Does the Abstract (Summary) clearly and accurately describe the content of the article? No

Is the problem significant and concisely stated? Yes

Are the methods described comprehensively? No

Are the interpretations and conclusions justified by the results? Yes

Is adequate reference made to other work in the field? Yes

## Manuscript Structure

Length of article is: Adequate

Number of tables is: Adequate

Number of figures is: Adequate

Please state any conflict(s) of interest that you have in relation to the review of this paper. None

Interest: Average

Quality: Below Average

Originality: Good

Overall: Average

Recommendation: Major Revision

## Comments

O objeto de estudo apresenta relevância uma vez que estuda a reabilitação Pós-Covid no contexto brasileiro.

Sugiro que os objetivos do estudo sejam melhor delimitados, com uma pergunta de pesquisa direcionada. As características dos pacientes atendidos pelo serviço não precisam ser um dos objetivos do estudo, mas dado de relevância, que deve estar contido na análise, para sustentar seus achados.

Indico o detalhamento da seção de métodos, clarificando de qual modalidade do sistema de saúde são os pacientes estudados (SUS, suplementar, ambos), características do serviço de saúde onde a pesquisa foi desenvolvida, os aspectos éticos e de consentimento empregados na pesquisa e, sobre a análise dos dados, identificar os desfechos de interesse e os fatores de associação.

Além disso, inserção da seção de introdução no resumo levará a melhor contextualização do objeto de estudo. 

